# MicroRNA-224 Targets SMAD Family Member 4 to Promote Cell Proliferation and Negatively Influence Patient Survival

**DOI:** 10.1371/journal.pone.0068744

**Published:** 2013-07-29

**Authors:** Yu Wang, Jianwei Ren, Yun Gao, Joel Z. I. Ma, Han Chong Toh, Pierce Chow, Alexander Y. F. Chung, London L. P. J. Ooi, Caroline G. L. Lee

**Affiliations:** 1 Division of Medical Sciences, Humphrey Oei Institute of Cancer Research, National Cancer Centre Singapore, Singapore, Singapore; 2 Department of Biochemistry, Yong Loo Lin School of Medicine, National University of Singapore, Singapore, Singapore; 3 Duke-NUS Graduate Medical School, Singapore, Singapore; 4 Department of Surgery, Singapore General Hospital, Singapore, Singapore; 5 Department of Surgical Oncology, National Cancer Centre Singapore, Singapore, Singapore; University of Hong Kong, Hong Kong

## Abstract

MicroRNA-224 (miR-224) is frequently over-expressed in liver and colorectal cancers. We and others have previously described the role of miR-224 over-expression in cell proliferation *in vitro* but we have yet to identify the relevant miR-224 direct target. In this study, we further demonstrated that miR-224 up-regulation promotes cell proliferation using both *in vitro* assays and *in vivo* tumor growth models. We systematically screened for high confidence miR-224 targets by overlapping *in silico* predicted targets from multiple algorithms and significantly down-regulated genes in miR-224-expressing cells from whole genome expression microarrays. A total of 72 high confidence miR-224 targets were identified and found to be enriched in various cancer-related processes. SMAD family member 4 (SMAD4) is experimentally validated as the direct cellular target through which miR-224 promotes cell proliferation. The clinical relevance of our experimental observations was supported by a statistically significant inverse correlation between miR-224 and SMAD4 transcript expression in tumor versus paired adjacent non-tumorous tissues from HCC patients (p<0.001, r = −0.45, R^2^ = 0.122). Furthermore, miR-224 up-regulation and SMAD4 down-regulation is significantly associated with poorer patient survival (p<0.05). In summary, miR-224/SMAD4 pathway is a clinically relevant pathway to provide new insights in understanding HCC. (191 words).

## Introduction

Aberrant microRNA expression features significantly in many cancers [1]. Many of these deregulated miRNAs are found to regulate crucial cellular targets such as Phosphatase and Tensin Homolog [2] and Signal Transducer and Activator of Transcription 3 [3]. miR-224 is one of the most commonly up-regulated miRNAs in HCC [4] and more recently also found to be over-expressed in other cancers such as colorectal cancer[5]–[7], cervical cancer [8] glioma [9] and breast cancer cells [10],suggesting elevated miR-224 expression may play a role in the general process of carcinogenesis.

The potential oncogenic property of miR-224 was demonstrated by various groups using *in vitro* cell line models to affect crucial cellular processes such as apoptosis [11], cell proliferation [11]–[13], cell migration and invasion [12]–[14]. We have previously reported apoptosis inhibitor 5 as a miR-224 direct target in the liver [11] while other groups have reported a handful of other miR-224 targets such as Smad family member 4 [13], Raf kinase inhibitor protein [10] and Type 1 iodothyronine deiodinase [15] in various other cell systems. However, a systematic examination of the direct cellular targets of miR-224 is important to understand its role in cancer. Furthermore, the *in vivo* function and the clinical significance of miR-224 over-expression in cancers such as HCC remain unclear.

In this study, we further characterized the role of miR-224 in promoting cell proliferation using both *in vitro* cell-based assays *in vivo* tumor explants in nude mice. We systematically screened for high confidence miR-224 putative targets by integrating *in silico* prediction and whole genome expression microarray data. SMAD family member 4 (SMAD4) was functionally validated as the clinically relevant cellular target through which miR-224 promotes cell proliferation. MiR-224 over-expression and SMAD4 down-regulation significantly correlates with a poorer overall survival in HCC patients.

## Materials and Methods

### Cell Lines, Mice and Patient Samples

Human colorectal HCT116 cells were cultured in modified McCoy’s 5A media (M4892,Sigma) supplemented with 10% fetal calf serum (04-001-1A/A, Bioindustries). Human hepatoma HepG2 cells were cultured in Dulbecco’s Modified Eagle Medium supplemented with 10% fetal calf serum. The cells were incubated at 37°C in a humidified atmosphere with 5% CO_2_. BALB/c nude mice were purchased from Biological Research Centre, Singapore. This study received ethical approval from National Cancer Centre Singapore (NCCS) Institutional Animal Care and Use Committee (IACUC: 2009/SHS/504A). HCC tumor and paired adjacent non-tumorous tissues from 100 hepatocellular carcinoma patients were obtained from the NCCS Tissue Repository with prior approval from the SingHealth Centralized IRB (SingHealth CIRB No: 2008/440/B).

### Whole Genome Expression Microarray

HCT116 cells were transfected with either 50 nM of Control Oligos or miR-224 precursors and harvested 24 hours post transduction. Total RNAs were extracted using mirVana™ microRNA Isolation Kit (AM1560, Ambion) and processed for Affymetrix Genechip (Affymetrix) hybridizations using U133A Genechips according to the instructions of the respective manufacturer. The hybridization signal on the chip was scanned and processed by GeneSuite software (Affymetrix). The microarray data can be retrieved from GEO database (GSE36020).

### *In Silico* Prediction of miR-224 Targets and Functional Annotation

miR-224 targets were predicted *in silico* using the miRecords [16]. Putative miR-224 targets were defined as those predicted by at least three independent algorithms. Gene list were mapped to gene ontology (GO) terms using DAVID [17], [18] and IDconverter [19].

### RT-qPCR and Western Blot Analysis

As previously described [3], [11], microRNA expression was determined using individual Taqman microRNA assay and transcript expression of target genes was measured with SYBR RT-qPCR (204143, Qiagen), normalized against endogenous control RNU48 and β-actin respectively. Protein expression was measured with Western blots and normalized against β-actin. miRNA assay ID, primer sequences and antibody ID are listed in Table 1.

**Table 1:** Small oligos, Taqman assays, primer sequences, antibodies.

Genes	Items	Sequence/Catalog ID
hsa-miR-224	Control Oligos	Ambion, AM17110
	Precursor	Ambion, PM12571
	Inhibitor	Ambion, AM12571
	Taqman Assay	ABI, 4373187
hsa-let-7d	Taqman Assay	ABI, 4373166
RNU48	Taqman Assay	ABI, 4373383
SMAD4	Primer	F: 5′-AGGATCAGTAGGTGGAATAG-3′
		R: 5′-TCTAAAGGTTGTGGGTCTGC-3′
	Antibody	Santa cruz, SC-56479
	siRNA	Sigma, SASI_Hs01_00207793
Beta-actin	Primer	F: 5′-ATGTTTGAGACCTTCAACACC-3′
		R: 5′-AGGTAGTCAGTCAGGTCCCGGCC-3′
	Antibody	Santa cruz, SC-1646

### 3′UTR Reporter Construct and Reporter Gene Assay

The 1313 base pairs (bps) wild type SMAD4 3′UTR (pSMAD4UTR-WT) and the miR-224 binding site mutant 3′UTR (pSMAD4UTR-MUT) were amplified from human placenta tissue and cloned downstream a β-galactosidase reporter gene (Fig. S1 in File S1). 3′UTR reporter assays were performed as previously described [3], [11].

### Stable Cell Line Generation

The pLemiR (miR-224) construct was purchased from Open Biosystems and the miR-224 insert was removed to serve as the control contruct. Both the pLemiR(miR-224) and the pLemiR(control) constructs were then transfected into HCT116 cells to generate stable clones under selection media containing 1 ug/ml of puromycin for 2 weeks. Clonal cells, which showed resistance to puromycin treatment and expressed turbo red fluorescence protein (tRFP), were further expanded and tested for miR-224 expression using RT-qPCR (Fig. S2 in File S1). Positive clones were grown in selection media for another 4 weeks to confirm stable expression. Two independent control clones (Clone 1 & 2) and two independent miR-224 expressing clones (Clone A & B) were used for subsequent functional assays.

### *In vitro* and *in vivo* Functional Assays

Cell proliferation was measured using the BrdU Cell Proliferation Kit (Calbiochem) accordingly to manufacturer’s instructions. Soft-agar colony forming assay were performed using two stable clones of miR-224 over-expressing cells and two stable clones of controls as previously described [20]. *In vivo* tumor growth was performed by subcutaneously injecting 5×10^6^ cells stably expressing miR-224 (clone A) or control (clone 1) into the left or right flanks of four BALB/C nude mice, respectively. The experiment was repeated using miR-224 expressing clone B paired with control clone 2 in three nude mice. Tumor size were measured on day 10, 20 and 26 post injection and tumor weight were measured on day 26 when the mice were sacrificed.

### Statistical Analysis

Analysis of Variance was used to compare sample means from three or more groups. Unpaired two-tailed t test was further performed to analyze the significance of differences between sample means between two groups from at least three independent experiments. Pearson correlation was used to analyze the relationship between the expression of miR-224 and SMAD4 in HCC patient samples. Kaplan-Meier survival analysis was used to analyze association of miR-224 & SMAD4 status and patient survival. Statistically significant tests were identified with p value less than 0.05.

## Results

### miR-224 Promotes Cell Proliferation *in vitro and in vivo*

**Figure 1:**
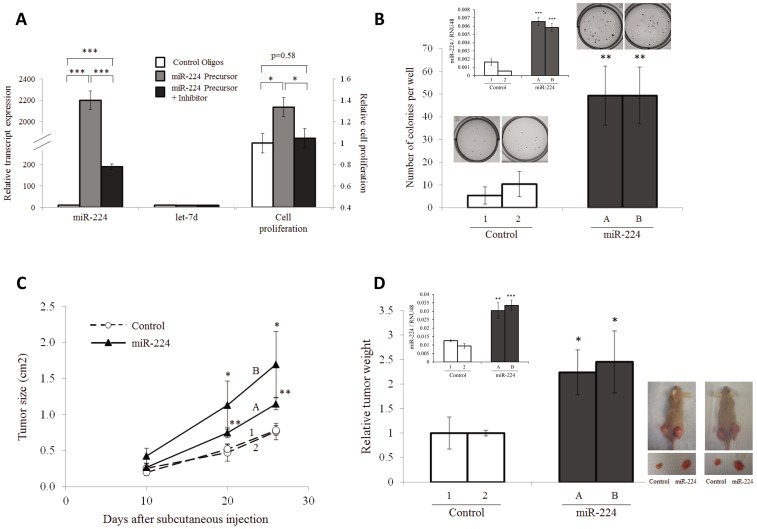
miR-224 promotes cell proliferation *in vitro* and *in vivo*. (**A**) Relative transcript expression of miR-224 (left panel) and non-specific let-7d expression (middle panel) measured using RT-qPCR and normalized against RNU48. Right panel shows the corresponding changes in cell proliferation measured with BrdU cell proliferation assay in cells transfected with either 50 nM of Control Oligos, miR-224 precursor or miR-224 precursor+inhibitor. (**B**) Colony formation from two stable clones of control (1 & 2) and two stable clones miR-224 expressing cells (A & B) grown on soft agar. Inset shows miR-224 expression and representative colony formation from the stable clones. Data in (A) and (B) were presented as Mean±SE from at least three independent experiments. (**C**) *In vivo* tumor growth from control or miR-224 expressing stable clones from (B) subcutaneously inoculated into BALB\c nude mice and plotted as tumor size versus time post subcutaneous injection. (**D**) *Left:* Average weight of tumors harvested from 4 mice each from clones 1 (control) and A (miR-224) or 3 mice each from clones 2 (control) and B (miR-224) at Day 26 post injection. Inset shows miR-224 expression in tumors from control or miR-224 expressing cells. *Right:* Photograph of representative mice inoculated with control or miR-224 expressing cells in the left and right dorsal area respectively. Data shown in (C) and (D) were presented as Mean±SD from four/three sacrificed nude mice per group respectively. * denotes p<0.05, ** denotes p<0.01 and *** denotes p<0.001.

miR-224 is found to be frequently over-expressed in hepatocellular carcinoma [4], [11] and colorectal cancer [5]–[7]. To understand the functional relevance of miR-224 over-expression in cancer, we initially over-express miR-224 in the human colon cancer cell line, HCT116, which exhibit low endogenous miR-224 expression (Fig. S3 in File S1). As shown in Figure 1A, ∼30% increase in cell proliferation (p<0.05, right panel, grey bar) was observed in cells over-expressing miR-224 (p<0.001, left panel, grey bar) compared to the Control Oligos transfected cells (white bar). Significantly, when miR-224 inhibitor and precursor were co-introduced into cells, the miR-224 over-expression was significantly inhibited (p<0.001, left panel, black bar) and cell proliferation was restored to a level similar to control cells (p = 0.58, right panel, black bar). These findings were then validated in the human hepatoma cell line HepG2 which exhibits moderate level of endogenous miR-224 levels (Fig. S3 in File S1). Similar to the observation in HCT116 cells, over-expression of miR-224 in HepG2 cells lead to a ∼35% increase in cell proliferation (p<0.01, Fig. S4 in File S1, rightmost panel, grey bar) while inhibition of endogenous miR-224 resulted in ∼20% decrease in cell proliferation (p<0.01, Fig. S4 in File S1, rightmost panel, black bar). The expression of unrelated let-7d remained unchanged, demonstrating the specificity of these small oligos in perturbing endogenous miR-224 level. Furthermore, triplicate experiments from two independent clonal cells stably expressing miR-224 (Clone A & B, p<0.001, Fig. 1B inset) formed ∼ five-fold more colonies on soft-agar compared to triplicate experiments from two independent control clones (Clone 1 & 2, p<0.01, Fig. 1B). Taken together, miR-224 over-expression promotes *in vitro* cell proliferation under both normal growth conditions and in soft-agar.

We proceeded to confirm our *in vitro* observations using an *in vivo* xenograft model in nude mice. Both miR-224 expressing (Clone A & B) and control stable cells (Clone 1 & 2) were able to form tumors 10 days after subcutaneous injection. Tumors from cells stably expressing miR-224 grew significantly faster than stable control cells (p<0.05, Fig. 1C). Furthermore, on Day 26, tumors harvested from miR-224-expressing stable cells were significantly heavier than the tumors from the control stable cells (p<0.05, Fig. 1D). Hence, over-expression of miR-224 not only promotes cell proliferation *in vitro* and but also tumor growth *in vivo.*

### miR-224 Potentially Targets Multiple Crucial Cellular Processes

**Figure 2:**
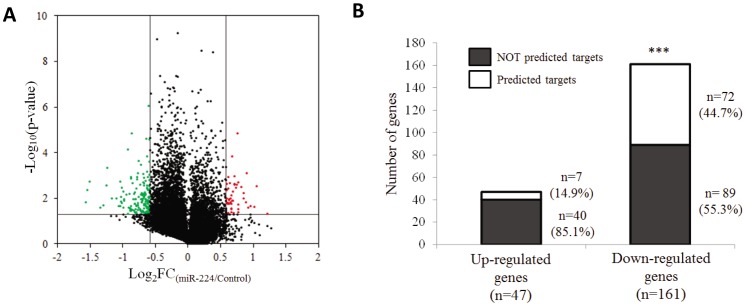
Putative miR-224 targets were significantly down-regulated in miR-224 expressing cells. (**A**) Volcano plot analysis of whole genome expression microarray data between cells transfected with miR-224 precursor and Control Oligos from three independent experiments. Differentially expressed genes that were both biologically and statistically significant were indicated in the two upper-lateral quadrant with absolute fold change >1.5 and p-value <0.05. Green spots indicated significantly down-regulated genes while red spots indicated significantly up-regulated genes in miR-224 expressing cells versus control cells. (**B**) Stacked barchart showing a significant enrichment of putative miR-224 targets in the significantly down-regulated genes in miR-224 expressing cells versus control cells. White box represents putative miR-224 targets predicted by at least three different algorithms while grey box represents the non-miR-224 target genes. Proportions of the predicted and non-predicted targets were indicated as percentages on the right of the intended box. P value is calculated from χ^2^ test.

As miRNAs affect cellular processes by regulating specific set of gene targets, whole genome expression microarray was employed to determine the profile of differentially expressed genes between miR-224 over-expressing cells and the control cells in three independent experiments. Greater than 3-fold more genes were found to be significantly down-regulated (161, |FC| >1.5, p<0.05) than up-regulated (47, |FC|<1.5, p<0.05), consistent with miRNAs being negative regulators of gene expression (Fig. 2A & Fig. 2B). Of the 161 genes that were down-regulated in miR-224 expressing cells, 72 (∼44.7%) were also predicted to be miR-224 direct cellular targets by at least three independent algorithms while only 7 of 47 (14.9%) up-regulated genes were predicted as miR-224 targets. These 72 significantly down-regulated genes which are also predicted as miR-224 targets by multiple algorithms are termed as high confidence miR-224 targets (miR-224 hc-targets) and further analyzed to elucidate the processes they affect. Apoptosis inhibitor 5 (API5), the only previously validated target of miR-224 in HCC [16], was found to be also amongst the 72 miR-224 hc-targets reaffirming the feasibility of this approach. As evident in Table 2, miR-224 hc-targets are enriched in cancer-related GO processes such as regulation of transcription, metabolic processes, apoptosis and signal transduction, suggesting that miR-224 over-expression can potentially affect multiple cellular processes.

**Table 2:** Gene ontology mapping of putative miR-224 targets which were significantly down-regulated in miR-224 expressing cells.

GO Term	GO ID	Genes Annotated to the GO term
Regulation of transcription DNA-dependent	GO:0006355	NFATC2IP, CDC42, SMAD4, ZNF395, C10ORF26, ZFP90, ZBTB43, TMF1, TFB1M, GCOM1
Metabolic process	GO:0008152	INSIG1, RDH10, SGPL1, HSDL1, TPD52, PFKFB3, PPAT
Apoptosis	GO:0006915	UBE4B, API5, SH3KBP1, SGPL1, RFFL
Signal transduction	GO:0007165	F2RL1, GRB10, CDC42BPA, RALA, GCC1
Protein phosphorylation	GO:0006468	CDC42BPA, SGK3, NUAK1
Proteolysis	GO:0006508	TPD52, ZFP90, ADAM17
Ubiquitin cycle	GO:0006512	UBE2D3, USP3, RFFL
Transport	GO:0006810	SLC37A4, TMED10, INSIG1
Intracellular protein transport	GO:0006886	FPNA6, RALA, RFFL
Cell-cell signalling	GO:0007267	SH3KBP1, ADAM17, GRB10
Protein transport	GO:0015031	TMED10, RALA, LIN7C
Protein ubiquitination	GO:0016567	FBXL5, SUMF1, UBE4B
Anti-apoptosis	GO:0006916	**API5**, SGK3
Cell proliferation	GO:0008283	INSIG1
Negative regulation of cell proliferation	GO:0008285	**SMAD4**

### SMAD4 is a Direct Cellular Target of miR-224

Amongst the 72 miR-224 hc-targets, SMAD4 is the only negative regulator of cell proliferation (Table 2), playing a role as an essential mediator of the transforming growth factor-beta (TGF-β) pathway, which acts as a potent inhibitor of cell proliferation [21]. We thus hypothesize that miR-224 can promote cell proliferation via targeting SMAD4.

**Figure 3:**
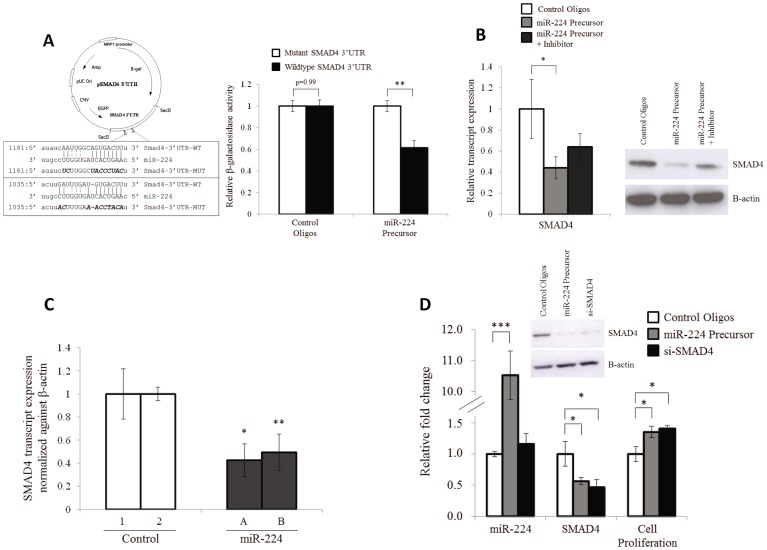
SMAD4 is a direct miR-224 target. (**A**) *Left:* Schematic diagram of the 3′UTR reporter constructs utilized to validate that the miR-224 targets the 3′UTR region of SMAD4, with the putative miR-224 binding sites and the mutated form highlighted in the box below. *Right:* Inhibitory effect of miR-224 on SMAD4 3′UTR examined through normalized β-galactosidase activity in cells co-transfected with Control oligos or miR-224 precursor and wild type SMAD4 3′UTR (Black bar) or mutant SMAD4 3′UTR reporter construct (White bar). (**B**) Relative SMAD4 transcript expression measured with RT-qPCR and normalized against β-actin (left panel) and SMAD4 protein expression measured by Western blotting in cells transfected with 50 nM of Control Oligos, miR-224 precursor or miR-224 precursor+inhibitor. (**C**) Relative SMAD4 transcript expression measured in the *in vivo* tumors arose from control (1 & 2) and miR-224 (A & B) stable clones expressing miR-224 (from Fig. 1D). (**D**) Relative fold change of miR-224 (Leftmost panel) and SMAD4 transcript expression (middle panel) and corresponding changes in cell proliferation measured with BrdU cell proliferation assay (rightmost panel) in cells transfected with either 50 nM of Control Oligos (white bar), miR-224 precursor (grey bar) or siRNA against SMAD4 (black bar). Inset shows the inhibition of SMAD4 protein by miR-224 precursor and siRNA against SMAD4. Data presented as Mean ± SE from three independent experiments. * denotes p<0.05, ** denotes p<0.01 and *** denotes p<0.001.

To experimentally validate SMAD4 as a miR-224 specific target, the wild type SMAD4 3′UTR (3′ untranslated region) as well as a mutant 3′UTR in which the two putative miR-224 binding sites were mutated, were cloned downstream a β-galactosidase (β-gal) reporter gene (Fig. 3A, left panel). Introduction of miR-224 precursors into cells containing wild-type SMAD4 3′UTR reporter construct resulted in significantly lower (p<0.01) β-gal activity compared to cells carrying the mutant SMAD4 3′UTR reporter construct in which the miR-224 binding sites are mutated (Fig. 3A, right panel). No significant difference was observed between cells carrying wild-type or mutant SMAD4 3′UTR reporter construct when Control Oligos were introduced. These data suggest that miR-224 specifically interacted with its putative binding sites along SMAD4 3′UTR to negatively regulate β-gal reporter gene activity.

The ability of miR-224 to modulate endogenous SMAD4 expression was then evaluated. Both SMAD4 transcript and protein expression were significantly reduced by ∼50% (p<0.05) in HCT116 cells transfected with miR-224 precursors and their expression can be partially reverted to normal by the co-transfection with both miR-224 precursor and inhibitor (Fig. 3B). No significant difference between miR-224-expressing and control cells was observed in the expression of transforming growth factor alpha (TGFA), beta (TGFB1 and TGFB2) and their receptors (TGFBR2 and TGFBR3), (Fig. S5 in File S1). Similarly, compared to control human hepatoma HepG2 cells, SMAD4 transcript expression was significantly reduced by ∼40% (p<0.05) in cells where miR-224 precursor was introduced and significantly increased by ∼60% (p<0.001) in cells where miR-224 inhibitor was introduced (Fig. S4 in File S1). Inhibition of SMAD4 expression was observed in tumors generated by miR-224 expressing stable clones (Clone A & B) compared to the control clones (Clone 1 & 2) (Fig. 3C). Taken together, our data demonstrates that SMAD4 is a direct miR-224 target.

To ascertain that SMAD4 is indeed a target through which miR-224 affects cell proliferation, SMAD4 expression was inhibited using siRNA that specifically targets SMAD4. As shown in Figure 3D, inhibition of SMAD4 expression in cells increased cell proliferation by ∼30% (p<0.05, black bar) which was phenotypically similar to the effect of overexpression of miR-224 by miR-224 precursors (p<0.05, grey bar). Taken together, these data strongly suggest SMAD4 as a miR-224 specific target through which miR-224 promotes cell proliferation.

### miR-224-SMAD4 is a Clinically Relevant Pathway in HCC

**Figure 4:**
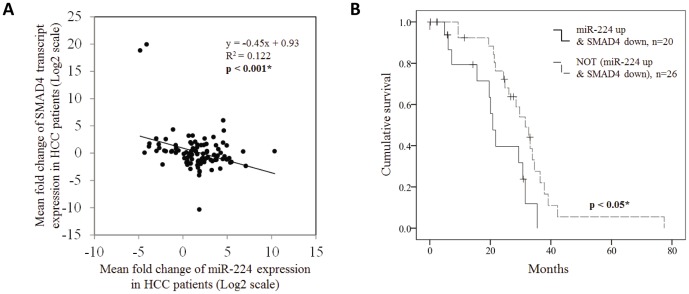
Up-regulation of miR-224 and down-regulation of SMAD4 expression is associated with poorer survival of HCC patients. (**A**) Scatter plots showing the statistically significant correlation in the relative transcript expression of miR-224 and SMAD4 (p<0.001) in the tumor versus paired adjacent non-tumor tissues from 100 HCC patients. Each spot represents data from one HCC patient presented in the Log_2_ scale and the linear regression line is depicted at the solid line. (**B**) Kaplan-Meier survival curve for 46 HCC patients classified based on whether HCC tumor showed miR-224 up-regulation (more than 2 fold) and SMAD4 down-regulation (more than 1.5 fold), compared to the paired adjacent non-tumor samples. Up-regulation of miR-224 and down-regulation of SMAD4 expression is significantly associated with poorer patient survival (log rank, p = 0.019).

We proceeded to evaluate the clinical relevance of our experimental observation by examining the expression of miR-224 and SMAD4 in the tumor and paired adjacent non-tumorous tissues from 100 HCC patients. A significant inverse correlation (p<0.001, r = −0.45, R^2^ = 0.122) was observed between the expression of miR-224 and SMAD4 in these HCC samples (Fig. 4A), suggesting SMAD4 as a clinically relevant miR-224 target in HCC. Although either miR-224 up-regulation or SMAD4 down-regulation alone did not show any significant association with overall patient survival (Fig. S6 in File S1), HCC patients with tumors which exhibits more than 2 fold up-regulated miR-224 and more than 1.5 fold down-regulated SMAD4 expression compared to the paired adjacent non-tumorous tissues were found to be significantly associated with poorer overall survival (p<0.05, Fig. 4B). Collectively, these data suggest that deregulation of miR-224 expression with subsequent deregulation of SMAD4 expression may serve as a prognostic biomarker predicting the patients’ survival outlook.

## Discussion

MicroRNA deregulation features significantly in tumorigenesis [1] and miR-224 is frequently observed to be over-expressed in a number of cancers. Recent evidence has linked miR-224 up-regulation in cancer with epigenetic reprogramming [22] and inflammation [23]. We and others have previously demonstrated that miR-224 up-regulation promotes cell proliferation [11]–[13] *in vitro*. Here we have further confirmed that high miR-224 expression also promotes tumor growth *in vivo*. To identify the cellular target through which miR-224 promotes cell proliferation we systematically screened for miR-224 putative targets by integrating multiple *in silico* miRNA-target gene prediction algorithms with experimental whole genome expression profiling of cells expressing miR-224. We identified a total of 72 high confidence miR-224 targets (hctargets), which are *in silico* predicted miR-224 targets with significantly reduced expression in miR-224 expressing cells (Fig. 2). Amongst these miR-224 hctargets, is API5, which was previously experimentally validated to be a specific target of miR-224 [11] providing confidence of our approach of identifying ‘genuine’ gene targets of miR-224. These miR-224 hctargets were predicted to potentially affect multiple cellular processes such as transcription, metabolism, cell proliferation and apoptosis, all of which play roles in tumorigenesis.

Notably, consistent with a recent report by Yao, *et al* who demonstrated that miR-224 is regulated by TGF-β/SMAD pathway and targets SMAD4 in mouse granulosa cells [13], our study also showed that in the human HCT116 cells, miR-224 indeed directly targets SMAD4, the central mediator of TGF-β pathway, to promote cell proliferation. Transforming growth factor beta (TGF-β) is a central regulator in chronic liver diseases that ranges from inflammation to cancer. Many studies have described a bipartite role of TGF-β with tumor suppressor functions that inhibits hepatocyte proliferation at early stages of liver cancer and oncogenic function that promotes invasion and metastasis at late stage of tumor progression [24]. Interestingly, miR-224 does not directly affect the expression of TGFα, TGFβ1 and TGFβ2 or their receptors (TGFβR2 and TGFβR3). miR-224 up-regulation was shown to be an very early event that precedes tumor transformation [22]. Our data suggests that in the early events of tumorigenesis, miR-224 up-regulation may suppress the antiproliferative function of TGF-β by down-regulating SMAD4 and attenuating the transcriptional response of TGF-β signalling transduction [25]. This is supported by that fact that SMAD4 knockout mice developed tumors throughout the gastrointestinal tract [26] and inactivation of SMAD4 has been shown to markedly attenuate the TGF-β-mediated antiproliferative response [27]. Although homozygous deletions or intragenic mutations account for up to 60% of the SMAD4 inactivation in pancreatic and colorectal carcinoma, no homozygous deletion and only 6% of intragenic mutations of SMAD4 were detected in HCC [25]. In this study, we provided evidence that SMAD4 could also be regulated by the aberrant expression of miR-224. Our observations, together with Yao *et al.* who demonstrated that miR-224 is regulated by TGF-β/SMAD pathway and targets SMAD4 in mouse granulosa cells [13] suggest the physiological role of miR-224 in the TGF-β signaling negative feedback loop. Curiously, at late stages of HCC, both miR-224 and TGF-β are shown to be over-expressed in HCC, promoting invasion and metastasis [12], [21]. However, it remains to be evaluated whether the underlying mechanisms are common or independent.

In summary, this study demonstrated that miR-224 up-regulation promotes cell proliferation *in vitro and in vivo*. Notably, deregulation of miR-224 together with its clinically relevant direct target gene, SMAD4 was found to be significantly associated with HCC patient survival status. Hence, miR-224-SMAD4 may represent a novel pathway for prognostic and therapeutic intervention.

## Supporting Information

File S1 **Figure S1, Strategy, primers and product of mutagenesis of SMAD4 3′UTR.** (**A**) Strategy for the generation of mutations at the miR-224 binding site of SMAD4 3′UTR. (**B**) Primers used for the generation of mutations at the miR-224 binding site of SMAD4 3′UTR. **Figure S2, Strategy to generate stable clonal cells expressing miR-224.** (**A**) Vector map of pLemiR construct in which tRFP, microRNA and puromycin resistance gene will be transcribed in a single long transcript. Positive clones will be resistant for puromycin and positive for RFP and the microRNA of interest. (B) A representative clone during selection. **Figure S3,** **Endogenous miR-224 expression in various cancer cell lines measured using Taqman RT-qPCR and normalized against RNU48 as endogenous control. Figure S4, Relative transcript expression of miR-224, non-specific let-7d and SMAD4 measured using RT-qPCR and normalized against RNU48 or beta-actin.** Right panel shows the corresponding changes in cell proliferation measured with BrdU cell proliferation assay in human hepatoma cell line HepG2 cells transfected with either 50 nM of Control Oligos, miR-224 precursor or miR-224 inhibitor. **Figure S5,** Relative transcript expression of transforming growth factor alpha (TGFA), beta (TGFB1 & TGFB2) and relevant receptors (TGFBR2 & TGFBR3) in HCT116 cells transfected with miR-224 precursors versus that with control oligos, as measured through cDNA microarrays. **Figure S6, Kaplan-Meier survival curve for 46 HCC patients classified based on (A) miR-224 status or (B) SMAD4 status.**
(PDF)
