# Supplementary material for: MicroRNA-224 Targets SMAD Family Member 4 to Promote Cell Proliferation and Negatively Influence Patient Survival
Source: PLoS One. 2013 Jul 29;8(7):e68744. doi: 10.1371/journal.pone.0068744 (PMC3726696; doi:10.1371/journal.pone.0068744)
Supplement: File S1 — Figure S1, Strategy, primers and product of mutagenesis of SMAD4 3′UTR. (A) Strategy for the generation of mutations at the miR-224 binding site of SMAD4 3′UTR. (B) Primers used for the generation of mutations at the miR-224 binding site of SMAD4 3′UTR. Figure S2, Strategy to generate stable clonal cells expressing miR-224. (A) Vector map of pLemiR construct in which tRFP, microRNA and puromycin resistance gene will be transcribed in a single long transcript. Positive clones will be resistant for puromycin and positive for RFP and the microRNA of interest. (B) A representative clone during selection. Figure S3, Endogenous miR-224 expression in various cancer cell lines measured using Taqman RT-qPCR and normalized against RNU48 as endogenous control. Figure S4, Relative transcript expression of miR-224, non-specific let-7d and SMAD4 measured using RT-qPCR and normalized against RNU48 or beta-actin. Right panel shows the corresponding changes in cell proliferation measured with BrdU cell proliferation assay in human hepatoma cell line HepG2 cells transfected with either 50 nM of Control Oligos, miR-224 precursor or miR-224 inhibitor. Figure S5, Relative transcript expression of transforming growth factor alpha (TGFA), beta (TGFB1 & TGFB2) and relevant receptors (TGFBR2 & TGFBR3) in HCT116 cells transfected with miR-224 precursors versus that with control oligos, as measured through cDNA microarrays. Figure S6, Kaplan-Meier survival curve for 46 HCC patients classified based on (A) miR-224 status or (B) SMAD4 status. (PDF) [file pone.0068744.s001.pdf]

Fig. S1

A

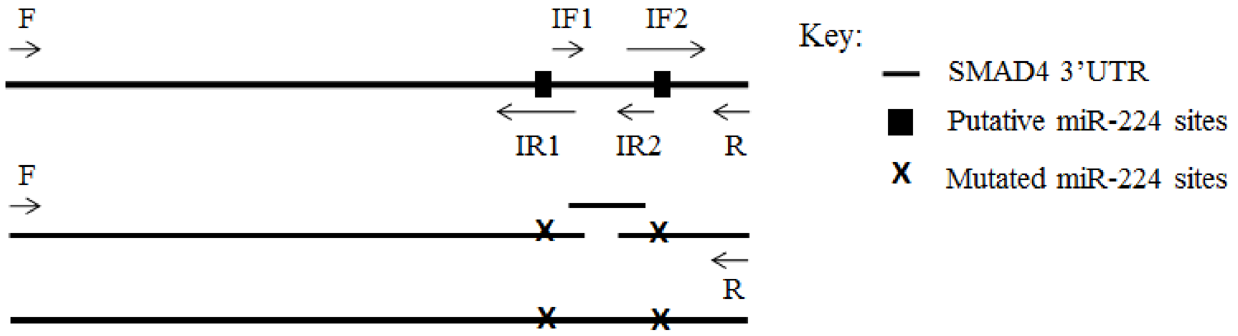

B

Primers	Sequences
Smad4_F	5'-GACTGACCGCGGTCTTTTACCGTTGGGGCCCTTAACC-3'
Smad4_R	5'-ATGGTTTTTGCAGTATCATTATTCA-3'
Smad4_IF1	5'-TTTTTGGTATAATGTTTAAATCATG-3'
Smad4_IR1	5'-CATGATTTAAACATTATACCAAAAATGTAGGTTTCAAAGTAAGTACAAAAATATCCAAACTACCTATTATG-3'
Smad4_IF2	5'-GCCCTTTGCCATCAATGATCATATCTCTTGGCTACCCTACTGTATAGAGAATTTAAGTAG-3'
Smad4_IR2	5'-GATATGATCATTGATGGCAAAGGGCT-3'

Strategy, primers and product of mutagenesis of SMAD4 3'UTR. (A) Strategy for the generation of mutations at the miR-224 binding site of SMAD4 3'UTR. **(B)** Primers used for the generation of mutations at the miR-224 binding site of SMAD4 3'UTR

Fig. S2

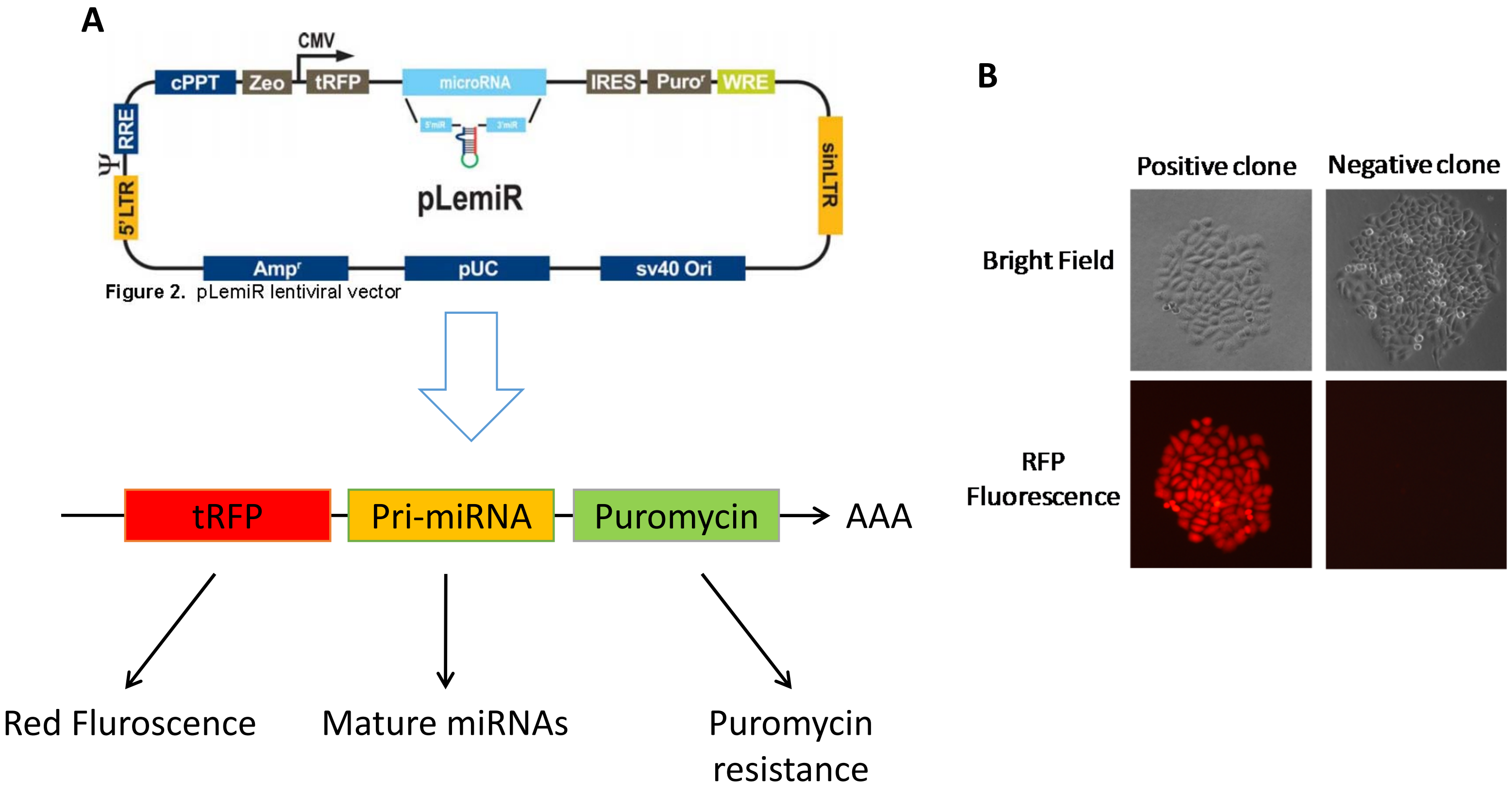

Strategy to generate stable clonal cells expressing miR-224. (A) Vector map of pLemiR construct in which tRFP, microRNA and puromycin resistance gene will be transcribed in a single long transcript. Positive clones will be resistant for puromycin and positive for RFP and the microRNA of interest. **(B)** A representative clone during selection.

Fig. S3

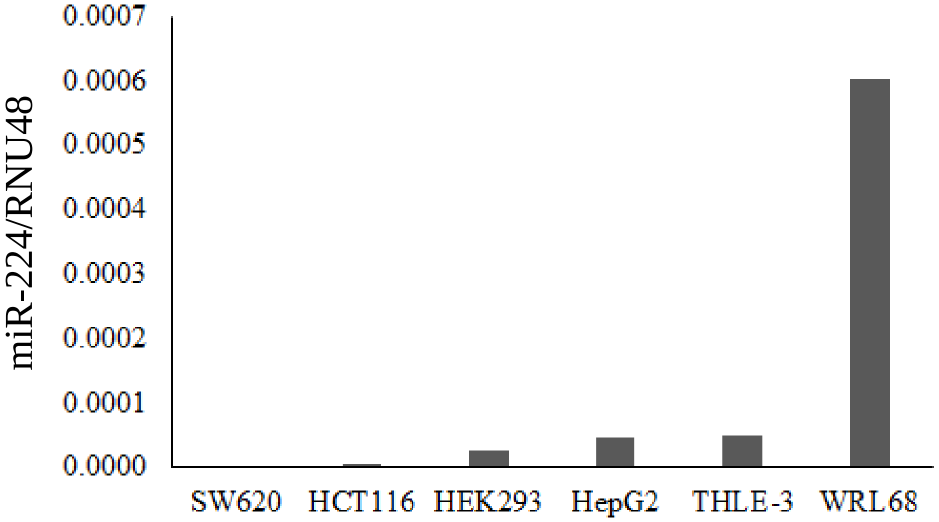

Endogenous miR-224 expression in various cancer cell lines measured using Taqman RT-qPCR and normalized against RNU48 as endogenous control.

Fig. S4

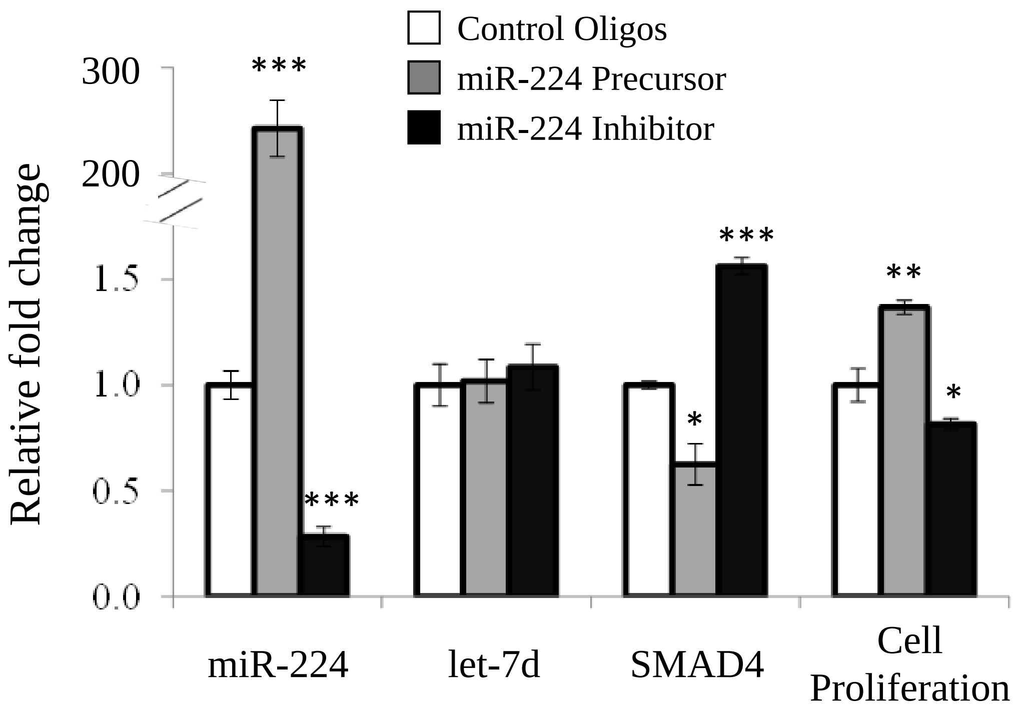

Relative transcript expression of miR-224, non-specific let-7d and SMAD4 measured using RT-qPCR and normalized against RNU48 or beta-actin. Right panel shows the corresponding changes in cell proliferation measured with BrdU cell proliferation assay in human hepatoma cell line HepG2 cells transfected with either 50 nM of Control Oligos, miR-224 precursor or miR-224 inhibitor.

Fig. S5

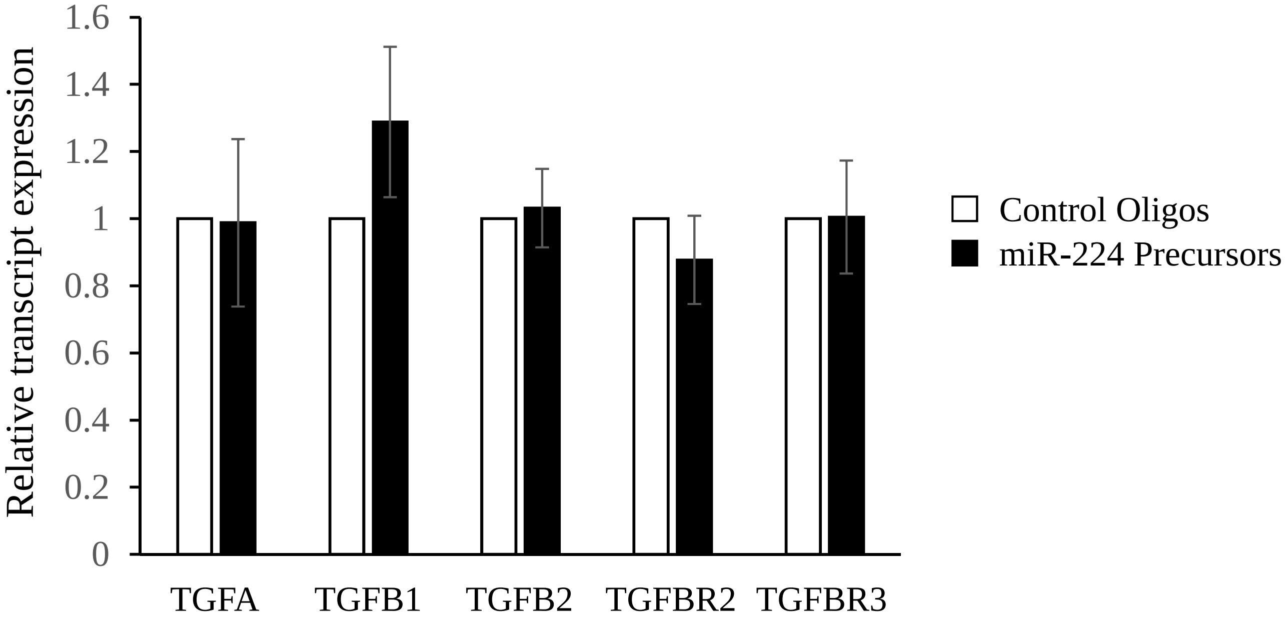

Relative transcript expression of transforming growth factor alpha (TGFA), beta (TGFB1 & TGFB2) and relevant receptors (TGFBR2 & TGFBR3) in HCT116 cells transfected with miR-224 precursors versus that with control oligos, as measured through cDNA microarrays.

Fig. S6

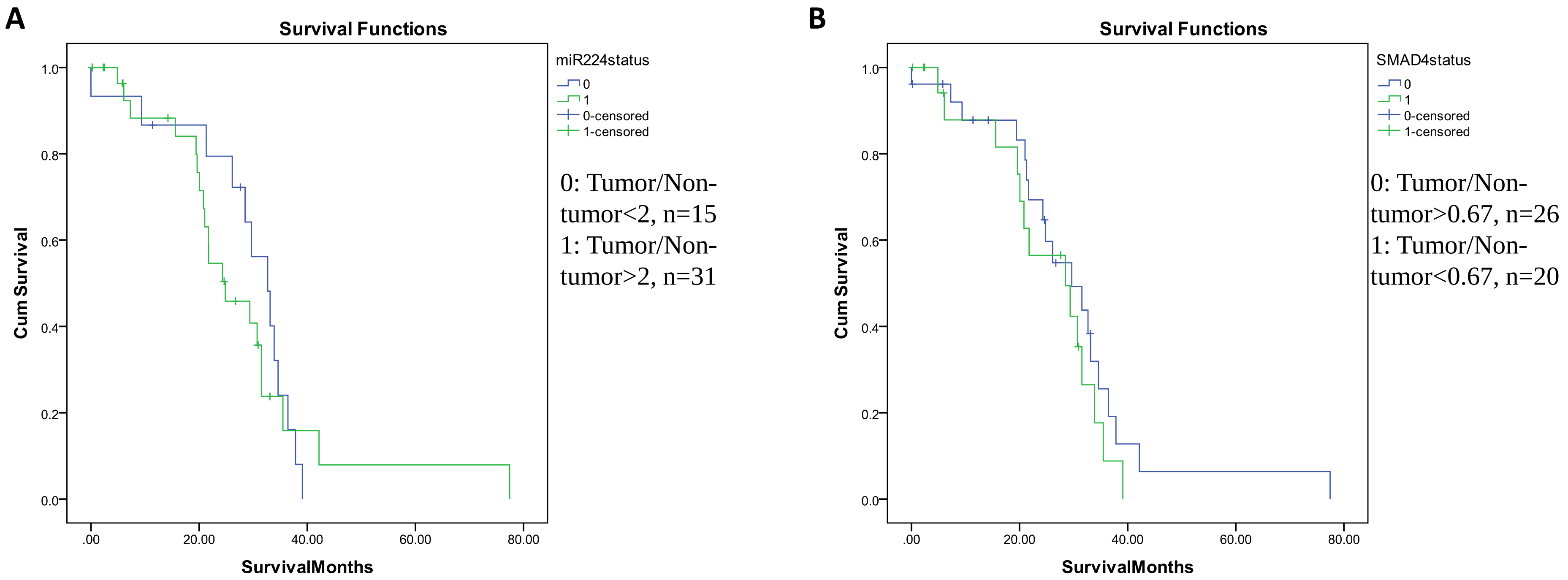

Kaplan-Meier survival curve for 46 HCC patients classified based on (A) miR-224 status or (B) SMAD4 status.
